# Supplementary material for: Exploring barriers and associated factors in the preparedness for cholera outbreak response in Public Health Centres of Addis Ababa, Ethiopia
Source: PLOS Glob Public Health. 2025 Sep 11;5(9):e0005087. doi: 10.1371/journal.pgph.0005087 (PMC12425226; doi:10.1371/journal.pgph.0005087)
Supplement: Attachment — Submitted filename: Response to Reviewer.docx [file pgph.0005087.s003.docx]

1. Conceptual Confusion between "Preparedness" and "Readiness". The paper consistently uses “preparedness” without defining it or distinguishing it from “readiness.” In public health, “preparedness” generally refers to long-term planning (e.g. training, protocols, resource allocation), while “readiness” refers to immediate operational ability to respond. By conflating or ignoring the distinction, the study may overestimate the system’s actual capability during an outbreak. The mention of adapting a checklist from the Ethiopian guideline for cholera “preparedness to assess readiness” creates ambiguity about the tool’s origin (Ethiopian government or WHO because sections mention the 14—point WHO checklist was used), and purpose, potentially conflating preparedness and response concepts. It remains unclear which questions assess preparedness, and which ones assess readiness, or at last operational definitions of the terms.

**Response-** Thank you for your thoughtful and constructive feedback on our manuscript. We greatly appreciate the time you took to review our work and provide valuable insights that have helped improve the clarity and precision of our study.

**Response to Comment #1**: Conceptual Clarity Between "Preparedness" and "Readiness"
We sincerely appreciate this observation. Upon reflection, we recognize that the distinction between preparedness (long-term planning) and readiness (immediate operational capacity) is crucial in public health emergency management. To address this, we have:

1. Defined both terms explicitly in the Methods section under *Operational Definitions*.
2. Clarified the origin of the assessment tool, it was adapted from both the Ethiopian national guideline and WHO checklist, ensuring alignment with both preparedness and readiness indicators.
3. Specified which components assessed preparedness vs. readiness in the data collection section (e.g., plans and training for preparedness; stockpiles and WASH for readiness).
4. Adjusted language in the Abstract and Discussion to reflect this distinction while acknowledging that the study’s primary focus was on structural preparedness.

These revisions ensure that our findings are interpreted with the appropriate context, evaluating both the foundational preparedness of health centers and their immediate readiness for outbreak response.

We hope these revisions address your concern satisfactorily. Please let us know if any further clarifications or adjustments would enhance the manuscript.

2. Over-Reliance on Binary Categorization

Health centers were labelled as either “prepared” or “not prepared” based on meeting 75% of checklist items. This threshold is arbitrary and lacks validation or justification in the paper. After all, a health center with 10/14 items may be nearly as capable as one with 11/14 but is considered unprepared - why did the authors choose binary categorization over all other options?

- It seems the 14 items were presumed to have equal weights in the assessment of preparedness, since no weighting of items was mentioned. Instance, can ORS availability and incinerator access be equal in outbreak response relevance?

**Response-**We sincerely appreciate your thoughtful critique regarding our binary classification approach and the equal weighting of checklist items. Your comments raise important methodological considerations that we would like to address with both transparency and gratitude for helping us improve our work.

Regarding the 75% Threshold:

We completely agree that the distinction between a health center scoring 10/14 versus 11/14 may not reflect meaningful operational differences in outbreak response capacity. Our choice of the 75% cutoff was primarily guided by:

The WHO's standardized approach for rapid health facility assessments in resource limited settings, where binary categorization is often pragmatically employed for programmatic decision-making

Ethiopia's national cholera guidelines which utilize similar threshold benchmarks for operational simplicity

However, we recognize this approach has limitations in sensitivity. In our revised manuscript, we will:

Explicitly acknowledge this as a study limitation

Regarding Equal Item Weighting:

You raise an excellent point about the differential importance of checklist items. While our tool treated all 14 items equally for consistency with the WHO's original framework, we absolutely agree that:

Certain items like ORS availability are clinically more urgent than others (e.g., incinerator access)

Weighting by item importance could provide a more accurate preparedness assessment. We will also acknowledge this in the limitation section of the revised manuscript.

3. Weakness in Statistical Analysis

Only one variable (presence of preparedness plan) was found statistically significant in multivariable analysis. Despite collecting many variables (e.g., training, previous cholera experience), the model explains little variance. This suggests either limited variability in preparedness scores, or inadequate power to detect meaningful associations.

Confidence intervals are wide, suggesting unstable estimates, for example, AOR of 6.88 (1.39–9.89) indicates lack of precision.

**Response-** Thank you for this insightful observation about our multivariable analysis. We appreciate the opportunity to clarify these important methodological considerations and have taken the following steps to address your concerns:

We acknowledge that only one variable (emergency preparedness plan) showed statistical significance in our final model. This likely reflects:

- The high baseline homogeneity in preparedness scores across facilities (as noted in Table 3, most centers scored similarly on key indicators like ORS availability [90.1%] and IV fluids [90.1%])
- Possible collinearity between predictor variables (e.g., facilities with preparedness plans were also more likely to have trained staff and stockpiles)

Regarding the wide confidence interval: You're absolutely correct that the wide CI for our primary association (AOR=6.88, 95% CI:1.39-9.89) indicates limited precision. This stems from:

- The relatively small number of "unprepared" facilities (n=45) creating instability in estimates
- The strong association between having a plan and being prepared (only 2 facilities with plans were unprepared), and we have Recommended future studies with larger samples of unprepared facilities to improve precision.

4. Methodological Concerns in Qualitative Component.

- Small sample size: Only 5 key informant interviews (KII) and 2 FGDs across 101 facilities, and no mention of the principle of saturation is included.

- Limited triangulation: No patient or community perspective is included, or at least some acknowledgement of this limitation.

**Response-** We sincerely appreciate your thoughtful critique regarding the qualitative component of our study. Your insights have helped us recognize important limitations that strengthen the transparency and rigor of our work. Here is how we have addressed your concerns:

Small Sample Size and Saturation:

You rightly note that our qualitative sample (5 KIIs and 2 FGDs) may appear limited relative to 101 facilities. We would like to clarify that:

Participant selection followed purposeful sampling to capture maximum variation (including sub-city representation, facility size, and responder roles)

While we did not explicitly mention saturation, thematic redundancy was achieved by the 4th KII and 2nd FGD, with no new themes emerging in final interviews

We have now added to the Methods: "Data collection continued until thematic saturation was reached, where no new concepts emerged in consecutive interviews"

Limited Triangulation:

We fully agree that incorporating patient/community perspectives would have enriched our understanding. This limitation reflects:

The study's primary focus on health system preparedness (facility-level capacity)

Ethical/logistical constraints during outbreak preparedness assessments

In response, we have:

Added this as a key limitation: "Findings reflect health worker and administrator perspectives; future research should incorporate community voices to triangulate barriers"

Included a recommendation for community-based participatory research in cholera preparedness

5. All the limitations that come with the study must be acknowledged, for example, consideration of community engagement. Public health preparedness includes community awareness, early warning systems, and risk communication. These are entirely omitted, though they are part of WHO’s preparedness indicators.

**Response:** Thank you for your thoughtful critique regarding the scope of our study’s preparedness assessment. You raise an excellent point; our facility-focused design did not capture critical community-level dimensions of cholera readiness, such as early warning systems, risk communication, and community engagement. We fully agree that these elements are essential to a holistic understanding of outbreak preparedness, as emphasized in WHO’s frameworks, and revised accordingly in the limitation section of the manuscript.

6. Overstatement in Conclusion. The conclusion claims "preparedness is relatively high," but only 55.4% of facilities met the criteria. This is not a majority worth characterizing as relatively high, especially for an urban capital with outbreak history.

**Response:** Thank you for this important critique regarding our language in the conclusion. We agree that characterizing 55.4% preparedness as "relatively high" requires more nuanced interpretation, and we have revised our framing accordingly in the revised manuscript.

7. Lack of Actionable Policy Recommendations. While the paper identifies barriers (financial, motivation, multisectoral coordination), no concrete solutions are proposed. Example: No costed plan, incentive strategies, or examples of successful multisectoral collaboration.

**Response-** Thank you for this valuable critique, and revised accordingly in the revised manuscript.
